# Development and Characterization of Recombinant Ovine Coagulation Factor VIII

**DOI:** 10.1371/journal.pone.0049481

**Published:** 2012-11-09

**Authors:** Philip M. Zakas, Bagirath Gangadharan, Graca Almeida-Porada, Christopher D. Porada, H. Trent Spencer, Christopher B. Doering

**Affiliations:** 1 Graduate Program in Molecular and Systems Pharmacology, Emory University, Atlanta, Georgia, United States of America; 2 Aflac Cancer and Blood Disorders Center, Department of Pediatrics, Emory University School of Medicine, Atlanta, Georgia, United States of America; 3 Wake Forest Institute for Regenerative Medicine, Winston-Salem, North Carolina, United States of America; Institut National de la Santé et de la Recherche Médicale, France

## Abstract

Animal models of the bleeding disorder, hemophilia A, have been an integral component of the biopharmaceutical development process and have facilitated the development of recombinant coagulation factor VIII (fVIII) products capable of restoring median survival of persons with hemophilia A to that of the general population. However, there remain several limitations to recombinant fVIII as a biotherapeutic, including invasiveness of intravenous infusion, short half-life, immunogenicity, and lack of availability to the majority of the world's population. The recently described ovine model of hemophilia A is the largest and most accurate phenocopy. Affected sheep die prematurely due to bleeding-related pathogenesis and display robust adaptive humoral immunity to non-ovine fVIII. Herein, we describe the development and characterization of recombinant ovine fVIII (ofVIII) to support further the utility of the ovine hemophilia A model. Full-length and B-domain deleted (BDD) ofVIII cDNAs were generated and demonstrated to facilitate greater biosynthetic rates than their human fVIII counterparts while both BDD constructs showed greater expression rates than the same-species full-length versions. A top recombinant BDD ofVIII producing baby hamster kidney clone was identified and used to biosynthesize raw material for purification and biochemical characterization. Highly purified recombinant BDD ofVIII preparations possess a specific activity nearly 2-fold higher than recombinant BDD human fVIII and display a differential glycosylation pattern. However, binding to the carrier protein, von Willebrand factor, which is critical for stability of fVIII in circulation, is indistinguishable. Decay of thrombin-activated ofVIIIa is 2-fold slower than human fVIII indicating greater intrinsic stability. Furthermore, intravenous administration of ofVIII effectively reverses the bleeding phenotype in the murine model of hemophilia A. Recombinant ofVIII should facilitate the maintenance of the ovine hemophilia A herd and their utilization as a relevant large animal model for the research and development of novel nucleic acid and protein-based therapies for hemophilia A.

## Introduction

Factor VIII (fVIII) is an essential glycoprotein procofactor within the intrinsic pathway of the blood coagulation cascade. In blood circulation, fVIII is non-covalently bound to von Willebrand factor (VWF) and is present at relatively low concentration (1 nM). Mutations in the *F8* gene often result in diminished or inactive plasma fVIII and are the molecular genetic cause of the monogenic, X-linked, bleeding disorder hemophilia A that affects approximately 1 in 7500 males worldwide. Current treatment is limited to intravenous infusion of plasma-derived or recombinant human fVIII (hfVIII) containing products. This therapy is only available to 30% of the world due to economic factors and requires multi-weekly injections to achieve prophylaxis, which must be maintained for the duration of the patients' life to avoid debilitating joint disease as well as life-threatening bleeding episodes. While gene therapy is being explored as a potential cure, additional research efforts are aimed at improving the therapeutic utility of recombinant fVIII.

Investigations into the biochemical properties of orthologous fVIII constructs have yielded insight into basic fVIII structure/function as well as translation into novel clinical agents. For example of the former, the characterization of recombinant murine factor VIII (mfVIII) revealed near complete stability at physiologic concentrations following thrombin activation [1]. Porcine fVIII (pfVIII) demonstrates 10 to 100-fold increased expression over hfVIII [2], [3], as well as decreased engagement of the endoplasmic reticulum-resident unfolded protein response [4]. Furthermore, Arruda and colleagues described the development and characterization of canine fVIII, which displays 3-fold higher specific activity than that of hfVIII and currently is utilized to manage bleeding in canine hemophilia A colonies [5]. As for the development of novel clinical agents, plasma derived pfVIII has historically been used in the treatment of patients with pre-existing inhibitors to hfVIII and recombinant B-domain deleted (BDD) pfVIII currently is in clinical trials. Likewise, human/porcine (hp) hybrid transgenes with high expression properties are being developed for clinical gene therapy [6].

A line of sheep presenting with hemophilia A recently was re-established and the pathology, clinical profile, and molecular genetics were described [7]. Ovine fVIII (ofVIII) possesses 86% amino acid sequence homology to hfVIII outside of the B-domain and possesses a similar domain structure (A1-A2-B-ap-A3-C1-C2) defined by internal sequence homology. The causative mutation was identified as a single nucleotide insertion resulting in frameshift and a premature stop codon in exon 14 similar to a mutation documented in a human patient with severe hemophilia A [8]. In preliminary studies, administration of hfVIII or hpfVIII corrected the bleeding phenotype in this model transiently, but invariably induced the formation of high-titer anti-fVIII inhibitory antibodies eventually leading to premature mortality. Moreover, transplantation of genetically-modified mesenchymal stem cells expressing a BDD pfVIII transgene in this model corrected phenotypic hemarthroses and spontaneous bleeds for several months, thus establishing the potential of the model for the development of novel therapeutics. The utility of this model as a research and development resource hinges on the ability to maintain colonies of these clinically fragile animals. Toward this goal, the cloning, expression, purification and biochemical characterization of recombinant BDD ofVIII are described in the current study.

## Materials and Methods

### Materials

The cloning and characterization of full-length ovine fVIII in the pUC57 vector has been described previously [7]. Phusion High Fidelity PCR MasterMix, PNGase, and all restriction enzymes were purchased from New England Biolabs (Ipswich, MA). All cell culture materials were purchased from Corning Inc. (Corning, NY). AIM V and DMEM/F12 media was purchased from Invitrogen (Carlsbad, CA). Citrated fVIII-deficient plasma and normal pooled human plasma (FACT) were purchased from George King Biomedical (Overland Park, KA). Activated partial thromboplastin reagent (aPTT) was purchased from Organon Teknika (Durham, NC). Monoclonal antibodies were provided by Dr. Pete Lollar (Aflac Cancer Center and Blood Disorders Service, Emory University, Atlanta, GA). Recombinant human thrombin was provided by Haematologic Technologies Inc. (Essex, VT). Desulfatohirudin was a generous gift from Dr. R. B. Wallis (Ciba-Geigy Pharmaceuticals) to our colleague Dr. Pete Lollar (Emory University, Atlanta, GA). SDS-PAGE precast gels were purchased from Bio-Rad (Hercules, CA). Polyethyleneimine was purchased from Polysciences, Inc. (Warrington, PA). A colony of exon 16-disrupted hemophilia A mice (E16 ^−/−^) was kept and maintained within the Emory Division of Animal Resources Pediatrics Facility.

### Generation of BDD OfVIII

Replacement of the ovine B-domain with an SQ linker containing a PACE/furin recognition sequence was conducted by SOE mutagenesis as described previously [1]. Primers for heavy chain (HC) and light chain (LC) were manufactured by Integrated DNA Technologies (Coralville, IA). HC forward: 5′- GAC CGG ATC GGA AAA CCT CTC GAG CCA CCA TGC ACA TCA AGC TCT GTA CCT GCC-3′; HC reverse: 5′-ATT CTG GGA GAA GCT CCT AGG TTC AAT GAC ATT GTT TTC ACT CAG CAG G-3′; LC forward: 5′-GTC ATT GAA CCT AGG AGC TTC TCC CAG AAT CCA CCA AGC TTG AAA CGC CAT CAA AGG-3′; LC reverse: 5′-AGT GGC AGG TGC TGC AGC GGC CGC CCT CAG TAC TGC TGC TGT GCC TCA C-3′. PCR amplification was conducted in the following cycles: 30 s at 98°C, 35 cycles of 10 s at 98°C and 30 s at 58°C, and annealing at 78°C for 13 minutes followed by 25°C hold. Amplified products were digested utilizing NotI, AvrII and XhoI restriction nucleases and separated using SeaKem® GTG® Agarose (Lonza; Rockland ME) gel electrophoresis. Digested fragments were purified using a QIAquick Gel Extraction Kit (Qiagen) and ligated using a T4 DNA Quick Ligase (Promega). The final construct was cloned into ReNeo mammalian expression vector using NotI and XhoI restriction sites. BDD ofVIII ReNeo was sequenced by Beckman Coulter Genomics (Danvers, MA) using overlapping primers spanning the entire transgene.

### Generation and Characterization of Stable OfVIII Expressing Clones

Naïve baby hamster kidney-derived (BHK-M) cells were transfected in 6-well plates with 1.5 µg/10^6^ cells of ReNeo mammalian expression plasmid encoding the respective fVIII transgene. Polyethylenimine was administered at a final concentration of 6 ng/ml in DMEM containing 10% FBS. Media was replaced at 24 h and expanded at 48 h to 10 cm plates in media containing 500 µg/ml G418 (Gibco, Grand Island, NY) and cultured for 10–14 days. Fifty to sixty-eight clones were selected and expanded. For determination of specific fVIII production rates, the clones were cultured for 24 h in serum free AIM V media and then counted by hemacytometer for normalization to units/10^6^ cells/24 hr. FVIII activity measurements were made by one-stage coagulation assay and linear regression analysis of clotting times against a pooled human plasma standard (FACT) using a ST art Coagulation Instrument (Diagnostica Stago, Asnieres, France). Geneticin-resistant clones that expressed fVIII below the limit of assay detection (0.01 units/ml) in 24 h were not included in the statistical analysis. From 35 antibiotic resistant clones with BDD ovine fVIII activity above measurable detection, the highest expression clone was selected for further study. Peak expression from this clone was 6 units/10^6^ cells/24 h.

### Purification of Recombinant OfVIII

Recombinant ofVIII was purified using a two-step ion exchange chromatography procedure as cited previously [2]. Expressing clones were expanded into 500 cm^2^ flasks in DMEM/F-12 growth media containing 10% FBS, 100 units/ml penicillin, and 100 µg/ml streptomycin. Cells at 60–70% confluency were washed 2× with 50 ml Dulbecco's phosphate-buffered saline (PBS) (Thermo Scientific) and re-fed with 125–150 ml AIM V serum free media. Media was collected every 24–48 h and replaced with equal volume of fresh AIM V. FVIII containing media was subjected to centrifugation at 2000× *g* for 10 min and the supernatant was frozen at −80°C in 0.05% sodium azide until time of purification. Media was thawed at 37°C and loaded onto a 5×20 cm SP-Sepharose High Performance column equilibrated to 0.15 M NaCl, 20 mM HEPES, 5 mM CaCl_2_, 0.01% Tween-80, pH 7.4 (HBST). The column was washed twice with equilibration buffer followed by 18% NaCl containing buffer prior to elution. Fractions were eluted over a linear 0.18–0.65 M NaCl gradient in HBST. Fractions containing fVIII were assayed for fVIII activity and activation quotient (AQ) and those with AQ values greater than 20 were pooled. The AQ assay was conducted using both one-stage and two-stage coagulation assays as described previously [1]. The activation quotient is defined as the ratio of fVIII activity measured by two-stage coagulation assay divided by the fVIII activity measured by the one-stage coagulation assay. Pooled material was diluted to 0.15 M NaCl in the HBST, applied to a Source Q HR5/5 FPLC column and eluted with a linear 0.2–0.65 M NaCl gradient. Fractions were assayed by one-stage coagulation assay, absorbance at 280 nm, and SDS-PAGE. Final AQ and specific activity measurements of pooled material were recorded following a freeze/thaw cycle at −80°C.

### SDS-PAGE Analysis and Mass Spectrometry

Polypeptides were resolved by 4–15% gradient SDS-PAGE and fixed with 50% methanol/10% glacial acetic acid prior to staining with GelCode Blue (Pierce, Rockford, IL). Deglycosylation was conducted according to manufacturer's directions (NEB). Briefly, 2 µg purified protein was activated with 2 units recombinant human thrombin at 37°C for 10 min, denatured in denaturation buffer supplied by the manufacturer at 95°C for 10 minutes, and deglycosylated by incubation with 500 units PNGase at 37°C for 1 hour. Confirmation of N-linked glycan location was carried out by tandem mass spectrometry. OfVIII was treated with PNGase as described above, purified via SDS-PAGE, and stained with coomassie blue. Excised bands were subjected to in-gel digestion (12.5 µg/ml trypsin). Extracted peptides were loaded onto a C18 column (100-µm inner diameter, 20 cm long, ∼300 nl/min flow rate, 1.9-µm resin from Dr. Maisch Gbmh, Ammerbuch-Entringen, Germany) and eluted during a 10–30% gradient (Buffer A: 0.1% formic acid, 1% ACN; Buffer B: 0.1% formic acid, 99.9% ACN). The eluted peptides were detected by Orbitrap (300–1600 m/z; 1,000,000 automatic gain control target; 500-ms maximum ion time; resolution, 30,000 full-width at half-maximum) followed by ten data-dependent MS/MS scans in the linear ion trap quadrupole (2 m/z isolation width, 35% collision energy, 5,000 automatic gain control target, 150-ms maximum ion time) on a hybrid mass spectrometer, LTQ Orbitrap XL (Thermo Fisher Scientific, San Jose, CA). The acquired tandem mass spectrometer (MS/MS) spectra were searched against and decoy-concatenated Ovis aries database (807 target proteins and 1 customized coagulation factor VIII sequence) from the NCBI RefSeq protein database project (version 54) using the Sorcerer-SEQUEST Algorithm version 4.04 (Sage-N Research, San Jose, CA) with differential modification of +0.984016 Da on Asn and +15.99492 on Met. Search results were filtered to 1% FDR and summarized by in-house programs, as described by Gozal et al [9]. Glycosylation sites were determined by differential analysis of peptide elution profiles and tandem mass spectrum between glycosylated and PNGase-F treated samples. In summary, ion chromatograms for peptides containing a matching asparagine modification mass shift were extracted from the PNGase-F treated sample. Only ions that have a noise-level chromatogram in the glycosylated sample were considered to be confidently matched to a glycopeptide.

### Immunoprecipitation

Purified polypeptide was activated with 1.4 µM thrombin, inactivated with 10 µM desulfatohirudin and incubated with mAb for 45 min at 37°C in M-PER (Thermo Scientific, Rockford, IL) with 150 mM NaCl. Protein G Agarose (KPL, Gaithersburg MD) was added and incubated at 37°C for one hour. Following centrifugation at 14,000× *g*, pellets were washed three times in 200 µl HBST followed by centrifugation at 14,000× *g* for 5 minutes. Protein G complexes were resuspended in 25 µl HBST and heated for 5 minutes at 95°C. Supernatant was loaded into 4–15% SDS-PAGE gel for analysis.

### Activated FVIII Decay Assay

Activated factor VIII (fVIIIa) was measured by chromogenic assay using purified human factor IXa, human factor X, and synthetic phospholipid vesicles as described previously [10]. Briefly, 20 nM ofVIII or hfVIII was activated with 100 nM human thrombin for 15 seconds at room temperature. Desulfatohirudin (150 nM) was added to stop the reaction and fVIII activity was measured at several time points.

### von Willebrand Factor (VWF) Binding

Binding of ofVIII to human VWF was determined by ELISA. Thermo Scientific Immulon 1B plates were coated with 50 µl of 6 mg/ml human VWF in buffer A (20 mM HEPES, 150 mM NaCl, 2 mM CaCl_2_, 0.05% Tween 20, 0.05% sodium azide) overnight at 4°C. Plates were washed twice with buffer A and blocked with 2% BSA in buffer A (blocking buffer). Plates were stored at 4°C until use. Human and ovine fVIII were diluted in blocking buffer and applied to VWF coated wells following two washes with buffer A. Plates were incubated for 1 h at room temperature, washed twice with buffer A, and incubated with 1 µg/ml of biotinylated monoclonal antibody 4F4 1B (generously provided by Dr. Pete Lollar, Emory University) in blocking buffer for 1 h at room temperature. Primary antibody incubation was followed by two washes with buffer A and the addition of streptavidin alkaline phosphatase (Jackson ImmunoResearch Labs, Inc., West Grove, PA) at 1∶15,000 in buffer A for 1 h at room temperature. Substrate activation was preceded by two final washes in buffer A. Colorimetric transmission was initiated by the addition of 80 µl *para*-Nitrophenylphosphate substrate (Bio-Rad; Hercules, PA) and recorded in kinetic mode as the change in *A*_405_ s^−1^. Rates were limited to a maximum optical density of 0.8 OD.

### Efficacy of OfVIII *In Vivo*

Hemostatic challenge was conducted as previously described [11] with alterations. All animal studies were reviewed and approved by the Emory University Institutional Animal Care and Use Committee (IACUC). Briefly, ofVIII was diluted in saline to a concentration of 300 units/kg mouse body weight. Eight to twelve week old E16 ^−^/^−^ hemophilia A mice were infused with either saline or ofVIII via tail vein injection. Immediately following injection, mice were anesthetized with 3.5% isoflurane at a flow rate of 1,000 ml/min for 5 minutes. Tails were placed into a 15 ml conical tube with 13 ml sterile saline at 37°C. Isoflurane was reduced to 2% at a flow rate of 500 ml/min and maintained for the duration of the experiment. At 15 min, tails were transected at 2 mm diameter as measured by wire gauge. In doing so, blood loss between mice is standardized to the diameter of tail vasculature rather than distal length. Blood was collected in a new, pre-weighed 15 ml conical containing 13 ml sterile saline at 37°C for 40 minutes and measured by change in mass and recorded by mg blood loss per gram body weight. The mean evaporative loss of two vials of 13 ml sterile saline at 37°C was used to correct for changes in mass of the efficacy treatments. One mouse injected with ofVIII displayed blood loss of 7 mg/g body weight, however, this likely was attributed to a technical error in transection.

## Results

### Heterologous Expression of Recombinant OfVIII

**Figure 1:**
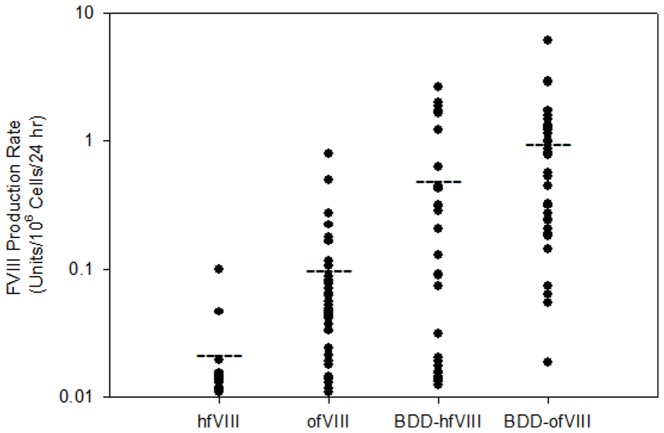
Expression of Recombinant Ovine and Human fVIII. BHK-M cells were stably transfected with full-length and BDD fVIII constructs and selected with geneticin. Individual colonies were expanded in 6-well plates and fVIII activity was measured by one-stage coagulation assay in serum-free media after 24 hr culture. Cell numbers were determined at the time of activity measurement and data was normalized to 10^6^ cells. The horizontal lines depict the mean values for each data set.

Recombinant fVIII can be synthesized in two distinct, but functional forms, the endogenous full-length form and the BDD form that displays enhanced expression due to a 1/3 reduction in transcript and transgene product size. Therefore, a recombinant BDD ovine fVIII (ofVIII) expression plasmid was designed that contained a PACE/furin recognition site (RHQR) within a 14 amino acid linker between A2 and ap-A3 domains similar to those described previously for other orthologous fVIII constructs [12], [13]. Expression of full-length and BDD human and ovine fVIII in serum-free media was compared using stably transfected BHK-M cells as described previously [1], [2], [6], [14] (Figure 1). Clones with measured activity production below 0.01 units/10^6^ cells/24 h (1% normal fVIII levels) were not included in statistical analysis. Clones expressing full-length ofVIII (n = 37) displayed significantly higher fVIII production rates, 0.0472 and 0.793 units/10^6^ cells/24 h (median and maximum, respectively), than clones expressing full length hfVIII (n = 15), 0.0142 and 0.1 units/10^6^ cells/24 h (median and maximum, respectively) (*P*<0.001, Mann-Whitney *U* test). Deletion of the B domain resulted in increased expression of both BBD ofVIII and BDD hfVIII over their full-length counterparts (*P*<0.001 for both comparisons, Mann-Whitney *U* test) with median expression levels of 0.783 and 0.091 units/10^6^ cells/24 h respectively. Again, the BDD ofVIII production rate was significantly greater than the BDD hfVIII rate (*P* = 0.005, Mann-Whitney *U* test). A 2.3 fold difference in expression between the top producing clones was observed with maximum clonal expression of BDD ofVIII measured at 6.12 units/10^6^ cells/24 h and BDD hfVIII at 2.63 units/10^6^ cells/24 h. The top producing BDD ofVIII clone was selected for production, purification and biochemical characterization of the final product.

### Purification and Biochemical Characterization of BDD OfVIII

**Figure 2:**
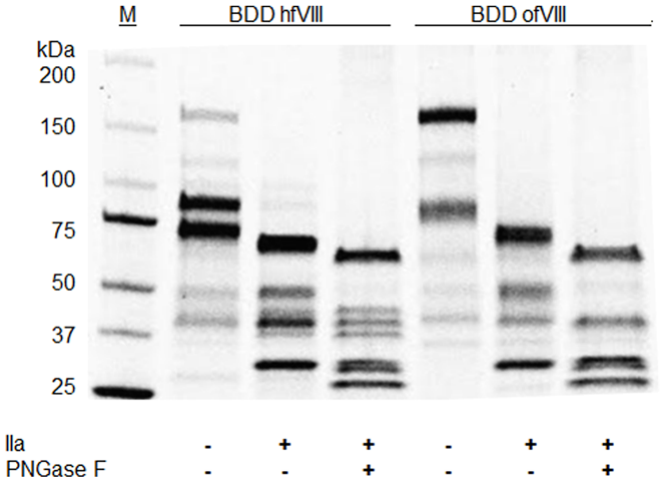
Biochemical Analysis of BDD OfVIII. Recombinant ofVIII (2 µg) ± thrombin and PNGase treatment was resolved by SDS-PAGE and visualized by Coomassie blue staining. A molecular weight ladder was used to determine the relative mobility of the polypeptides.

Three independent expression and purification experiments were conducted although two were at smaller scale. The average specific activity determined from these independent preparations was 2,516±503 U/nmol or 15,130±3,030 units/mg (mean ± standard deviation) polypeptide. Specific activity was calculated by absorbance at 280 nm and an estimation of the molar extinction coefficient based on known tyrosine, tryptophan, and cysteine content [15]. In one experiment, approximately 20,000 units of BDD ofVIII was collected in 5.4 L of serum-free media and purified using a two-step ion-exchange chromatography procedure (Table 1). The process yield was 25% and the final material had a specific activity of 3,050 units per nanomole (18,300 units/mg). One potential source of contamination that can affect dramatically the specific activity determination is the presence of activated fVIII (fVIIIa) in the final preparation. To address this issue, activation quotient (AQ) analysis of the peak fractions and final pooled material was performed. The activation quotient serves as a quality control metric for purified recombinant fVIII with an acceptable value being >20 and typically <80. A low AQ signifies the presence of fVIIIa in the material that can artificially inflate the activity measured in the one-stage coagulation assay and affect the accuracy of the specific activity determination. The AQ of the final BDD ofVIII preparation was 55. Furthermore, overall purity of >95% (fVIII polypeptides) was confirmed by SDS-PAGE analysis (Figure 2). The purified material from this preparation was utilized for all of the *in vitro* and *in vivo* characterization experiments presented herein.

**Table 1:** Purification of BDD OfVIII.

Sample	Vol. (ml)	A_280_	Total A_280_	Activity (U/ml)	Units	Units/A_280_	AQ	% Yield	Fold Pur.
Media	5,350	1.53	8,186	3.78	20,223	2.47	28	100	1
SP-Sepharose pool	35	0.415	14.5	353.4	12,370	853	49	61	345
Source Q pool	2.4	0.211	0.506	2,149	5,158	10,193	55	25.5	4,126

### Heterodimeric Structure, Glycan Analysis, and Thrombin Proteolysis of BDD OfVIII

**Figure 3:**
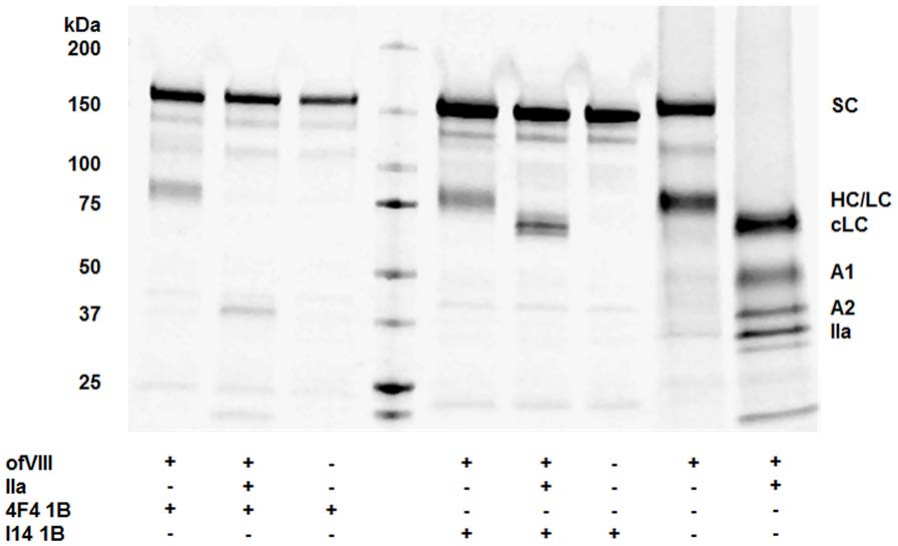
Discrimination of the BDD OfVIII Heavy and Light Chains. Immunoprecipitation using domain specific MAbs was performed by incubation with ofVIII in the presence and absence of thrombin. Heavy and light chains were dissociated using M-PER lysis buffer supplemented with 150 mM NaCl prior to MAb addition. FVIII heavy chain was precipitated with 4F4 1B, an A2 domain-specific mAb, and light chain was precipitated with I14 1B, a C2 domain-specific mAb. MAbs incubated with vehicle served as negative controls.

FVIII circulates in plasma as a heterodimer of heavy and light chains associated in a metal ion facilitated, non-covalent manner. Typically, these two large polypeptides readily can be resolved upon visual inspection following SDS-PAGE. Unique to BDD ofVIII, the heavy and light chain polypeptides display similar relative mobility upon SDS-PAGE (Figures 2 and 3). When compared to the respective BDD hfVIII sequence, the ovine A1 and A2 domain sequences contain five and three amino acid residue deletions, respectively. This results in a predicted 1.1 kDa decrease in the overall size of the BDD ofVIII heavy chain. The overall size of both human and ovine light chains is identical based on *in silico* prediction as well as empirical SDS-PAGE analysis. Eight asparagine residues can be identified as potential sites of N-linked glycosylation based on *in silico* analysis: Asn-41; Asn-213; Asn-239; Asn-582; Asn-1720; Asn-1810; Asn-2118; Asn-2270. Of these sites, Asn-582 and Asn-2270 are not predicted to actually contain oligosaccharides. Consistent with these predictions, in many distinct human fVIII preparations, it has been demonstrated that Asn-582 is not glycosylated [16]. Furthermore, treatment of thrombin-activated ofVIII with PNGase F did not alter the mobility of the ovine A2 domain thus supporting the prediction that Asn-582 is not glycosylated in BDD ofVIII (Figure 2). However, PNGase F treatment did affect a change in relative mobility of the ofVIII A1 and activated light chain fragments compared to BDD hFVIII, which is consistent with the prediction of additional domain-specific N-linked glycans within both regions. Asn-213 is not present in the human sequence, but is conserved in canine and porcine while Asn-1720 is to-date a uniquely described potential glycosylation site, although this site is not supported by mass spectrometry. MS/MS analysis supports with high confidence the glycosylation of Asn-41, Asn-1810, and Asn-2118. There remains evidence of glycosylation of Asn-213 and Asn-239, however, limitations in the resolution require further investigation. A2 and C2 domain specific MAbs were used to elucidate mobility of heavy and light chains independently. Purified BDD ofVIII was incubated with either 4F4 1B or I14 1B MAbs in a solution known to dissociate the fVIII heavy and light chains of fVIII heterodimers. Consistent with prediction of identical relative mobility of the BDD ofVIII heavy and light chain polypeptides, independent precipitations of BDD ofVIII material with heavy and light chain-specific MAbs yielded polypeptide species of equal mobility (Figure 3). Immunoprecipitations also were conducted in the presence of thrombin to verify domain-specific MAb interaction.

### Decay of Activated OfVIII

**Figure 4:**
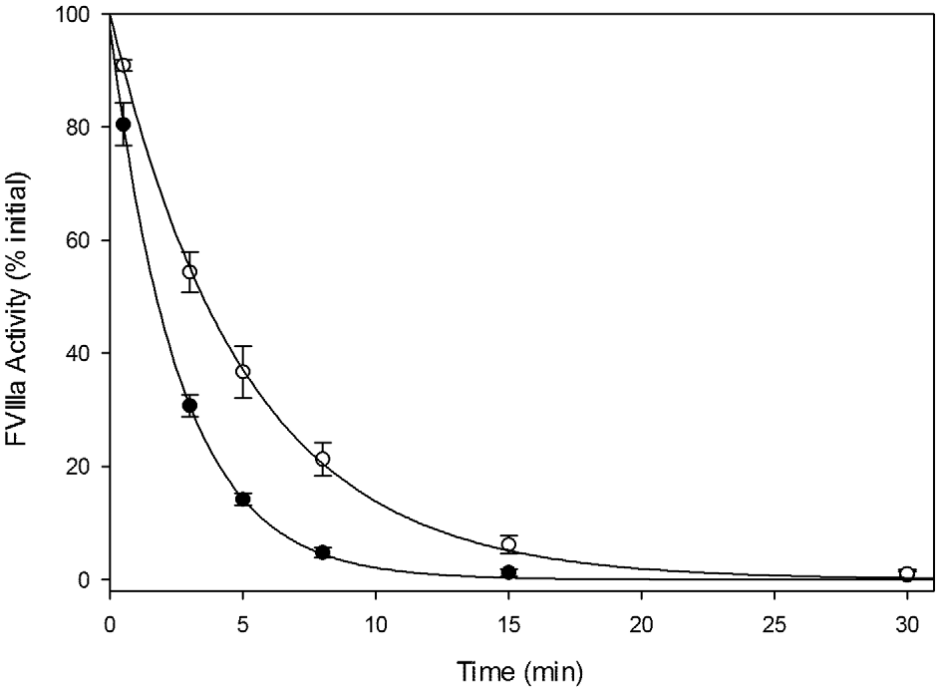
Thrombin-Activated Decay Rate of OfVIIIa. Human (closed circle) and ovine (open circle) fVIIIa decay was measured by chromogenic Xase assay in which 20 nM fVIII was activated with thrombin and then stopped with desulfatohirudin. Activated fVIIIa in complex with phospholipid vesicles, activated factor IXa, and factor X was measured at 0.5, 3, 5, 8, 15, and 30 minutes to determine residual fVIIIa activity. Half-lives of 1.8±0.09 and 3.5±0.37 minutes were calculated for human and ovine fVIIIa, respectively. Data shown represents the percent of initial activity by semi-log extrapolation to time  = 0. Regression analysis revealed Pearson correlation coefficients of 0.999 for both treatments.

When activated, fVIIIa serves as a cofactor for factor IXa, which executes the proteolytic cleavage of factor X into its activated form. Dissociation of the A2 domain from the A1/A3-C1-C2 heterodimer results in loss of pro-coagulant fVIII activity and can be measured indirectly by the generation of factor Xa in a purified system [10], [15], [17], [18], [19]. BDD ofVIII and BDD hfVIII were activated with thrombin and residual activity was measured over 30 minutes (Figure 4). Similar to previous reports, hfVIIIa displayed a mean (± sample standard deviation) half-life of 1.8±0.087 min [1], [2], [5] while the ofVIIIa half-life was prolonged significantly to 3.5±0.37 min (*P* = 0.001, Student's *t* test).

### VWF Binding

**Figure 5:**
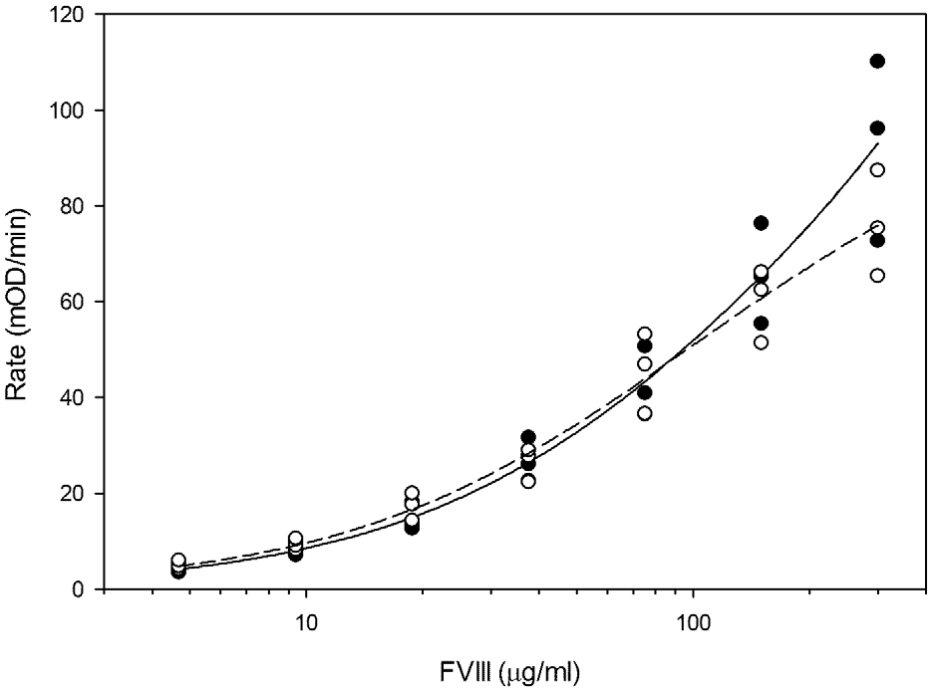
BDD OfVIII Binding to VWF. Kinetic ELISA was conducted using human VWF to capture human (closed circles) or ovine (open circles) fVIII. Plates were coated with 50 µl of 6 mg/ml human VWF and blocked with 2% BSA. Monoclonal A2 domain fVIII antibody 4F4 1B was added to each well and colorimetric transmission was activated with *para*-nitrophenylphosphate substrate following streptavidin alkaline phosphatase. Data shown are the mean of three independent experiments ± sample standard deviation.

VWF is a plasma glycoprotein that performs many roles in the hemostatic system. One of which is to stabilize fVIII in circulation through non-covalent association. VWF circulates as non-uniformly sized multimers composed of individual 270-kDa monomers. Each VWF monomer is capable of 1∶1 stoichiometric binding with fVIII. Upon proteolytic activation by thrombin or factor Xa, fVIII dissociates from VWF and is available to participate with factor IXa and Ca^2+^ in the formation of the tenase complex on a negatively charged phospholipid surface. In the absence of fVIII/VWF association, e.g. due to genetic deficiency of VWF or mutation of the fVIII/VWF binding sites, circulating fVIII levels are severely reduced and pathogenic bleeding often present phenotypically. The ability of ofVIII to bind human VWF, since a source of ovine VWF was unavailable, was assessed using an ELISA developed specifically for this study. Briefly, fVIII was captured by human VWF pre-adsorbed to a plate and detected using a MAb with an A2 domain epitope, which was demonstrated to possess equivalent affinity for human and ovine fVIII (Data not shown). Using this assay, BDD ofVIII and hfVIII displayed indistinguishable binding to VWF at physiologically relevant concentrations (Figure 5).

### *In Vivo* Efficacy of OfVIII

**Figure 6:**
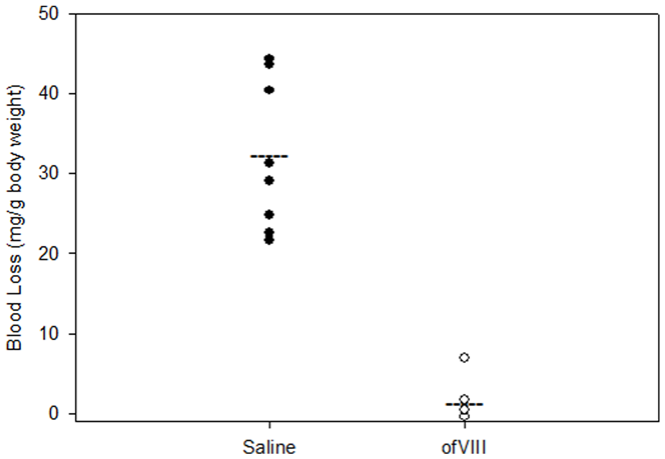
In Vivo Efficacy of OfVIII in Hemophilia A Mice. Hemophilia A mice were injected with either 100 µl saline or 300 U/kg ofVIII in 100 µl sterile saline via tail vein injection (n = 8). After 15 min, bleeding challenge was induced via tail transaction at 2 mm diameter. Blood was collected in pre-weighed vials of 13 ml sterile saline at 37°C. Blood loss was calculated and displayed as mg/g body weight. Mean blood loss for saline and ofVIII treatments were 32.2±9.37 and 1.15±2.57 mg/g body weight, respectively (*P*<0.001; Mann-Whitney *U* test).

In order to demonstrate functionality of BDD ofVIII to restore the blood coagulation *in vivo*, hemophilia A mice were injected with either saline or ofVIII at a dose of 300 units/kg, which was determined previously to restore plasma fVIII activity to near endogenous murine levels (2.9 units/ml for C57Bl/6 mice in the colony at Emory University) [1]. Following fVIII or saline administration, a hemostatic challenge was induced via a tail transection at the 2 mm diameter position of the tail and blood loss was measured over a 40-minute period. Hemophilia A mice injected with saline alone demonstrated a mean (± sample standard deviation) blood loss of 32.2±9.4 mg/g body weight (Figure 6). In contrast, mice injected with ofVIII demonstrated a mean blood loss of 1.15±2.57 mg/g body weight, which was significantly less than controls (*P*<0.001; Mann-Whitney Rank Sum Test) and consistent with complete correction of the bleeding phenotype in this model.

## Discussion

Innovation in hemophilia A treatment has benefited significantly from the use of animal models of the disease and the study of orthologous fVIII molecules. For example, hemophilia A biopharmaceuticals continue to be hindered by low-level cellular production whether they be produced endogenously (plasma-derived fVIII), heterologously (recombinant fVIII), or following gene transfer into the patient (gene therapy). Secretion of fVIII is observed at 2–3 orders of magnitude lower than glycoproteins of similar size, including homologous factor V [20]. This is attributed to inefficient mRNA expression, protein mis-folding, and engagement of the unfolded protein response, and subsequent inefficient and rate-limiting transport from the endoplasmic reticulum to Golgi apparatus. Recently, we identified or generated orthologous and bioengineered fVIII constructs, respectively, that display enhanced secretion efficiency over BDD hfVIII. Specifically, certain pfVIII sequences were identified and shown to improve the efficiency of fVIII secretion by 10–100-fold. FVIII constructs containing these high expression porcine fVIII sequences now have been demonstrated to outperform BDD hfVIII in heterologous expression systems [21], [22], [23] and in preclinical gene therapy studies [6], [14], [24]. Therefore, a rational design approach for novel hemophilia A therapeutics has arisen out of the discovery of species-specific differentials in certain properties such as the rate of biosynthesis, half-life, antigenicity and immunogenicity [1], [2], [3], [6], [10], [25], [26].

Disregarding the B-domain, the majority of fVIII orthologs contain greater than 80% amino acid identity to human fVIII. Despite this primary sequence similarity, numerous unique properties have been characterized and utilized toward bioengineering improved fVIII constructs. During re-establishment of a line of hemophilia A sheep, a new fVIII ortholog was cloned and characterized with the potential for discovering novel biochemical characteristics, while additionally providing a life-saving therapeutic for the ovine hemophilia A colony. In the current study, bioengineering, heterologous expression and biochemical characterization of ofVIII are described. Both recombinant full length and BDD ofVIII were shown to be expressed at greater levels than the equivalent hfVIII constructs in a BHK-M based heterologous expression system. These constructs were not modified beyond deletion of the B domain and the inclusion of a PACE/furin linker between the A2 and A3 domains as previously described [12], [13]. The biosynthesis levels observed for full length and hfVIII are comparable to previously published reports [2], [6], [14]. It was possible to purify significant quantities of BDD ofVIII using the same two-step ion exchange purification procedure that previously was described for recombinant BDD human, porcine and murine fVIII. OfVIII was purified to near homogeneity and shown to harbor specific activity higher than has been described previously for BDD human or murine fVIII [1], [2]. The purified product displayed AQ values indicative of very little to no contamination of the product with fVIIIa, which would artificially inflate the specific activity measurement. Therefore, it is concluded that the specific activity of BDD ofVIII is approximately 1.5 and 3 fold higher than recombinant BDD human and murine fVIII, respectively.

BDD ofVIII shares 86% amino acid identity to BDD hfVIII with eight amino acid deletions in the heavy chain. Analysis of N-linked glycosylation patterns with the use of PNGase F endoglycosidase revealed a greater mobility shift in the activated light chain and A1 domain of ofVIII than was observed for BDD hfVIII. Both the A1 and A3 domains of ofVIII have predicted N-linked glycosylation sites not present in hfVIII, the former also being present in canine, porcine, and murine fVIII. The results of mass spectrometry analysis of BDD ofVIII do not support the presence of N-linked glycosylation at Asn-1720, and an explanation for the differential mobility of the ovine fVIII light chain remains elusive. However, one possibility is that the processed glycans vary in size and composition thus accounting for the observed discrepancy. Similar to what was described for recombinant canine fVIII by Arruda and colleagues, the majority of secreted BDD ofVIII is maintained as single chain despite the presence of a consensus RHQR PACE/furin recognition site [5]. Entirely unique to ofVIII is the observation that there is not clear separation of the heterodimeric heavy and light chains observed upon SDS-PAGE. The chains are resolved upon immunoprecipitation of each polypeptide independently using domain-specific MAbs under conditions where the heterodimeric fVIII is dissociated. Following thrombin proteolysis, the A1 domain, A2 domain, and the activated light chain could be immunoprecipitated specifically. The biochemical basis of this observation is not yet understood. It seems likely that the amino acid deletions in the heavy chain coupled with potentially larger glycans bound to the light chain alter the relative mobilities in opposite, but converging directions. However, the predicted relative changes in molecular mass for these structural disparities (approximately 1 kDa for missing residues), may not be sufficient to account for this observation entirely. The differences in other post-translational modifications (e.g. O-linked glycosylation and tyrosine sulfation) may contribute to the diminished mobility of ovine light chain, but have not yet been characterized.

Following activation by thrombin, fVIII assumes a heterotrimeric structure with the A2 subunit being in weak association with the A1/A3-C1-C2 heterodimer, the latter of which is stabilized by coordination of a metal ion [18], [27]. Under physiologic concentrations of approximately 1 nM, the fVIII heterotrimer is thermodynamically unstable and the A2 subunit dissociates from the molecule with a half-life of 2 min for hfVIIIa. Dissociation of the A2 subunit results in the loss of fVIII coagulant function as demonstrated through identification of specific hemophilia A mutations that operate through this mechanism [28]. Although the physical factors directly attributing to A2 subunit dissociation are unclear, it has been shown that instability of the A2 domain association does not factor into one-stage coagulation assay fVIII activity measurements. As a result, measures of specific activity are independent of decay and must be due to factors other than A2 domain stability. However, in the presence of thrombin, mutations in the A1 or A2 domain resulting in diminished stability will show a reduced activity in the two-stage coagulation as compared to the one-stage assay and reduced ability to achieve hemostasis. Previously, we characterized the thrombin-activated decay of recombinant human, porcine, murine and hybrid fVIII molecules [1], [2]. Compared to those orthologs, ofVIII displays an intermediate half-life of 3.7 min, which is almost twice that of hfVIIIa but 0.5-fold that of pfVIIIa and <0.1-fold that of mfVIIIa. Increases in animal size and severity of potential thrombotic effects due to pressure differentials or vascular characteristics may provide an explanation for evolutionarily altered stability of fVIIIa within these species. Tight regulation of fVIII activity must be maintained to prevent unwarranted thrombotic events, as well as to allow cessation of bleeding events upon vessel injury.

In order to test the efficacy of BDD ofVIII, E16^−/−^ hemophilia A mice were challenged with a tail transection after the intravenous administration of 300 units/kg ofVIII compared to saline. In an attempt to normalize physical hemodynamic-related properties due to vasculature size, transections were made at 2 mm diameter as opposed to a fixed distance from the distal end of the tail [29], [30], [31]. Furthermore, to prevent the false appearance of phenotypic recovery at low time points due to non-fibrinogenic platelet aggregation at the site of transection, mice were observed for 40 minutes post-challenge. Blood loss was reduced dramatically and phenotypic correction was observed through recombinant BDD ofVIII administration. Based on this result, we believe it practical to test the efficacy of BDD ofVIII in hemophilia A sheep and further assess the propensity for BDD ofVIII induced inhibitor formation (i.e., immunogenicity).

Assuming a typical weight of 75 kg, an adult hemophilia A sheep would require approximately 1.5 mg ofVIII per administration using an estimated therapeutic dose of 300 units/kg translated from the data obtained in the current study using hemophilia A mice. Due to the demonstrated bio-production characteristics of ofVIII shown herein, prophylactic treatment of hemophilia A sheep would be similar in product requirements to that of humans with severe hemophilia and may not be practical due to manufacturing and economic constraints, e.g. the typical cost of prophylactic severe hemophilia A care in the U.S. is $200,000–300,000 per patient per yr. However, a 5 kg neonatal lamb would require only 80 µg per administration. If effective, this post-natal treatment regimen may be complimented by gene therapy trials to measure the safety, efficacy, and immunogenicity of novel gene therapy strategies in neonatal sheep, which would greatly enhance the value of this model in biomedical research.

As has been observed in some canine hemophilia A lines [5], [32], hemophilia A sheep develop inhibitors to recombinant human fVIII following parenteral infusion [7]. Hemophilia A sheep possess a premature stop codon in exon 14 caused by a frameshift mutation and are believed to be an accurate phenocopy of severe hemophilia A in humans. If different in any demonstrable manner, the sheep model may display a more severe bleeding phenotype as well as higher inhibitor incidence. The physiology and clinical phenotype is mirrored in ovine and human, and the former model eliminates the requirement of scale up estimation of treatment dosages. Maintaining the ovine hemophilia A colony requires intensive effort and extensive resources. Thus, without adequate validation, this model likely will be lost as a testing ground for the efficacy and immunogenicity of novel hemophilia A biotherapeutics and gene therapy applications. The development and characterization of recombinant ofVIII should facilitate the validation which in turn will enhance the value and utility of this unique large animal disease model.

## References

[1] Doering CB, Parker ET, Healey JF, Craddock HN, Barrow RT, et al (2002) Expression and characterization of recombinant murine factor VIII. Thromb Haemost 88: 450–458. 12353075

[2] Doering CB, Healey JF, Parker ET, Barrow RT, Lollar P (2002) High level expression of recombinant porcine coagulation factor VIII. J Biol Chem 277: 38345–38349. 12138172 10.1074/jbc.M206959200

[3] Doering CB, Healey JF, Parker ET, Barrow RT, Lollar P (2004) Identification of porcine coagulation factor VIII domains responsible for high level expression via enhanced secretion. J Biol Chem 279: 6546–6552. 14660593 10.1074/jbc.M312451200

[4] Brown HC, Gangadharan B, Doering CB (2011) Enhanced biosynthesis of coagulation factor VIII through diminished engagement of the unfolded protein response. J Biol Chem 286: 24451–24457. 21606503 10.1074/jbc.M111.238758 PMC3129224

[5] Sabatino DE, Freguia CF, Toso R, Santos A, Merricks EP, Kazazian HH Jr, Nichols TC, Camire RM, Arruda VR (2009) Recombinant canine B-domain-deleted FVIII exhibits high specific activity and is safe in the canine hemophilia A model. Blood 114: 4562–4565. 19770361 10.1182/blood-2009-05-220327 PMC2925478

[6] Doering CB, Denning G, Dooriss K, Gangadharan B, Johnston JM, et al (2009) Directed engineering of a high-expression chimeric transgene as a strategy for gene therapy of hemophilia A. Mol Ther 17: 1145–1154. 19259064 10.1038/mt.2009.35 PMC2835206

[7] Porada CD, Sanada C, Long CR, Wood JA, Desai J, et al (2009) Clinical and molecular characterization of a re-established line of sheep exhibiting hemophilia A. J Thromb Haemost 8: 276–285. 19943872 10.1111/j.1538-7836.2009.03697.x PMC2826196

[8] Vidal F, Farssac E, Altisent C, Puig L, Gallardo D (2000) A novel mutation (2409delT) in exon 14 of the factor VIII gene causes severe haemophilia A. Hum Hered 50: 266–267. 10782022 10.1159/000022928

[9] Gozal YM, Seyfried NT, Gearing M, Glass JD, Heilman CJ, et al (2011) Aberrant septin 11 is associated with sporadic frontotemporal lobar degeneration. Mol Neurodegener 10.1186/1750-1326-6-82. 10.1186/1750-1326-6-82 PMC3259087 22126117

[10] Lollar P, Parker ET, Fay PJ (1992) Coagulant properties of hybrid human/porcine factor VIII molecules. JBiolChem 267: 23652–23657. 1429706

[11] Spencer HT, Denning G, Gautney RE, Dropulic B, Roy AJ, et al (2011) Lentiviral vector platform for production of bioengineered recombinant coagulation factor VIII. Mol Ther 19: 302–309. 21081907 10.1038/mt.2010.239 PMC3034847

[12] Seidah NG, Chretien M (1997) Eukaryotic protein processing: endoproteolysis of precursor proteins. Curr Opin Biotechnol 8: 602–607. 9353231 10.1016/s0958-1669(97)80036-5

[13] Lind P, Larsson K, Spira J, Sydow-Backman M, Almstedt A, et al (1995) Novel forms of B-domain-deleted recombinant factor VIII molecules. Construction and biochemical characterization. Eur J Biochem 232: 19–27. 7556150 10.1111/j.1432-1033.1995.tb20776.x

[14] Dooriss KL, Denning G, Gangadharan B, Javazon EH, McCarty DA, et al (2009) Comparison of factor VIII transgenes bioengineered for improved expression in gene therapy of hemophilia A. Hum Gene Ther 20: 465–478. 19222367 10.1089/hum.2008.150 PMC2828624

[15] Pace CN, Vajdos F, Fee L, Grimsley G, Gray T (1995) How to measure and predict the molar absorption coefficient of a protein. Protein Sci 4: 2411–2423. 8563639 10.1002/pro.5560041120 PMC2143013

[16] Bihoreau N, Veillon JF, Ramon C, Scohyers JM, Schmitter JM (1995) Characterization of a recombinant antihaemophilia-A factor (factor VIII-delta II) by matrix-assisted laser desorption/ionization mass spectrometry. Rapid Commun Mass Spectrom 9: 1584–1588. 8652881 10.1002/rcm.1290091524

[17] Fay PJ, Haidaris PJ, Smudzin TM (1991) Human factor VIIIa subunit structure. Reconstruction of factor VIIIa from the isolated A1/A3-C1-C2 dimer and A2 subunit. J Biol Chem 266: 8957–8962. 1902833

[18] Lollar P, Parker CG (1990) pH-dependent denaturation of thrombin-activated porcine factor VIII. J Biol Chem 265: 1688–1692. 2295651

[19] Lollar P, Parker ET (1991) Structural basis for the decreased procoagulant activity of human factor VIII compared to the porcine homolog. J Biol Chem 266: 12481–12486. 1905722

[20] Pittman DD, Tomkinson KN, Kaufman RJ (1994) Post-translational requirements for functional factor V and factor VIII secretion in mammalian cells. J Biol Chem 269: 17329–17337. 8006042

[21] Selvaraj SR, Scheller AN, Miao HZ, Kaufman RJ, Pipe SW (2012) Bioengineering of coagulation factor VIII for efficient expression through elimination of a dispensable disulfide loop. J Thromb Haemost 10: 107–115. 22044596 10.1111/j.1538-7836.2011.04545.x PMC3290727

[22] Miao HZ, Sirachainan N, Palmer L, Kucab P, Cunningham MA, et al (2004) Bioengineering of coagulation factor VIII for improved secretion. Blood 103: 3412–3419. 14726380 10.1182/blood-2003-10-3591

[23] Ward NJ, Buckley SM, Waddington SN, Vandendriessche T, Chuah MK, et al (2011) Codon optimization of human factor VIII cDNAs leads to high-level expression. Blood 117: 798–807. 21041718 10.1182/blood-2010-05-282707

[24] Ide LM, Iwakoshi NN, Gangadharan B, Jobe S, Moot R, et al (2010) Functional aspects of factor VIII expression after transplantation of genetically-modified hematopoietic stem cells for hemophilia A. J Gene Med 12: 333–344. 20209485 10.1002/jgm.1442

[25] Parker ET, Doering CB, Lollar P (2006) A1 subunit-mediated regulation of thrombin-activated factor VIII A2 subunit dissociation. J Biol Chem 281: 13922–13930. 16513639 10.1074/jbc.M513124200

[26] Parker ET, Healey JF, Barrow RT, Craddock HN, Lollar P (2004) Reduction of the inhibitory antibody response to human factor VIII in hemophilia A mice by mutagenesis of the A2 domain B-cell epitope. Blood 104: 704–710. 15073030 10.1182/blood-2003-11-3891

[27] Lynch CM, Israel DI, Kaufman RJ, Miller AD (1993) Sequences in the coding region of clotting factor VIII act as dominant inhibitors of RNA accumulation and protein production. HumGene Ther 4: 259–272. 10.1089/hum.1993.4.3-259 8338874

[28] Pipe SW, Eickhorst AN, McKinley SH, Saenko EL, Kaufman RJ (1999) Mild hemophilia A caused by increased rate of factor VIII A2 subunit dissociation: evidence for nonproteolytic inactivation of factor VIIIa in vivo. Blood 93: 176–183. 9864159

[29] Chavez CL, Keravala A, Chu JN, Farruggio AP, Cuéllar VE, Voorberg J, Calos MP (2012) Long-Term Expression of Human Coagulation Factor VIII in a Tolerant Mouse Model Using the φC31 Integrase System. Hum Gene Ther 23: 390–398. 22077817 10.1089/hum.2011.110 PMC3327602

[30] Kumaran V, Benten D, Follenzi A, Joseph B, Sarkar R, Gupta S (2005) Transplantation of endothelial cells corrects the phenotype in hemophilia A mice. J Thromb Haemost 3: 2022–2031. 16102109 10.1111/j.1538-7836.2005.01508.x

[31] Yadav N, Kanjirakkuzhiyil S, Ramakrishnan M, Das TK, Mukhopadhyay A (2012) Factor VIII can be synthesized in hemophilia A mice liver by bone marrow progenitor cell-derived hepatocytes and sinusoidal endothelial cells. Stem Cells Dev 21: 110–120. 21480781 10.1089/scd.2010.0569

[32] Littlewood JD, Barrowcliffe TW (1987) The development and characterisation of antibodies to human factor VIII in haemophilic dogs. Thromb Haemost 57: 314–321. 3116702

